# A combination of circulating tumor cells and CA199 improves the diagnosis of pancreatic cancer

**DOI:** 10.1002/jcla.24341

**Published:** 2022-03-25

**Authors:** Junliang Chen, Huaitao Wang, Lei Zhou, Zhihao Liu, Xiaodong Tan

**Affiliations:** ^1^ 85024 Department of General Surgery Shengjing Hospital of China Medical University Shenyang China

**Keywords:** CA199, circulating tumor cell, CTC, early diagnosis, pancreatic cancer

## Abstract

**Background:**

Early diagnosis of pancreatic ductal adenocarcinoma (PDAC) is difficult due to the lack of effective screening tests. CA199, the standard biomarker for PDAC management, is not sufficiently reliable for early diagnosis. This prospective study aimed to evaluate whether circulating tumor cells (CTCs) could complement or perform better than CA199 in determining PDAC.

**Methods:**

A total of 168 blood samples were collected from 80 patients with PDAC, 32 patients with acute pancreatitis, 22 patients with benign pancreatic masses, and 34 healthy donors. CTCs were detected by a novel system combining negative enrichment with immunostaining and fluorescence in situ hybridization (NE‐imFISH). Next, ROC curves and AUC analyses were conducted to assess diagnostic abilities of CA199, CTCs, and the combination of the two biomarkers in PDAC.

**Results:**

CTCs were stained as CD45–/DAPI+/CEP8 ≥3. With 2 CTCs/3.2 ml as the cut‐off value, the sensitivity/specificity of the CTC number was 0.76/0.94, which was comparable to that of CA199 (0.78/0.83; Delong test *p* = 0.3360). Improved performance was achieved through a logistic regression model integrating CA199 and CTC number (AUC_CTC+CA199_ = 0.95, AUC_CA199_ = 0.80, AUC_CTC number_ = 0.85; Delong test *p*_vs_. _CA199_ < 0.0001 and *p*_vs_. _CTC number_ = 0.0002). CTC subtype was inferior to CTC number as a diagnostic marker (AUC_CTC subtype_ = 0.73; Delong test *p*_vs_. _CTC number_ < 0.0001).

**Conclusion:**

The dual‐marker panel consisting of CA199 and CTC number can significantly improve upon the diagnostic performance of CA199 alone, highlighting the promising clinical utilization as an effective strategy for PDAC surveillance.

## Graphical abstract

In this prospective study, the NE‐imFISH strategy was employed to identify CTCs in 80 PDAC patients and 88 controls, which were characterized by CD45–/DAPI+/CEP8 ≥3. As individual biomarkers, CTC number was comparable to CA199 in detecting PDAC. Of note, the dual‐marker panel consisting of CA199 and CTC number can significantly improve upon the diagnostic performance of CA199 alone.

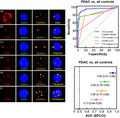

## 1 INTRODUCTION

Pancreatic ductal adenocarcinoma (PDAC), the fourth‐leading cause of cancer‐related death with an overall 5‐year survival of <8%, is predicted to rank second by 2030, exceeding mortality from breast and colorectal cancer.^1^, ^2^, ^3^, ^4^ Due to the insidious onset and aggressive nature, approximately 80%–85% of PDAC patients are diagnosed at advanced stages, losing the opportunities of radical resection.^2^, ^5^ Developing effective and convenient measures to improve early diagnosis is an urgent problem that remains to be addressed. Imaging tests, including CT, MRI, ultrasonography, are commonly used for diagnosis, but they have limited abilities to depict small tumors with similar signals to surrounding tissues.^6^, ^7^ Although Endoscopic ultrasound‐guided fine‐needle aspiration (EUS‐FNA) can provide preoperative biopsy, it is an invasive method accompanied by various complications, including pancreatitis, hemorrhage, infection, perforation, and needle tract seeding.^8^, ^9^ Thus, a minimally invasive, early diagnostic test with high sensitivity and specificity is highly desirable. Currently, Carbohydrate antigen 199 (CA199) is the sole serum biomarker that has received approval from the US Food and Drug Administration (FDA) for routine management of PDAC. This standard marker has 60%–90% sensitivity and 68%–91% specificity for diagnosis, with false‐negative results observed in Lewis antigen‐negative patients and false‐positive results caused by benign diseases, such as pancreatitis and cholangitis.^10^ Because of its modest diagnostic performance, there is a vital necessity to identify effective biomarkers to either complement or perform better than CA199.

Circulating tumor cells (CTCs) were first discovered in the peripheral circulation of a metastatic breast cancer patient by Prof. Ashworth in 1869.^11^ As cancer cells that detach from primary or metastatic tumors and spread through the bloodstream, CTCs may lead to tumor metastasis and relapse.^4^, ^12^ Owing to the technical difficulties, it took almost 150 years to establish a method to isolate 1 CTC from 5 billion erythrocytes and 10 million leukocytes present in the 1 ml of whole blood.^3^, ^4^ CellSearch^®^ is the only assay approved by FDA in 2004 for CTC detection in metastatic breast, prostate, and colorectal cancer, but the detection rate is only 21%–45% in PDAC patients.^3^, ^13^, ^14^, ^15^, ^16^, ^17^, ^18^, ^19^ The low efficiency of this platform is attributed to the dependence on epithelial cell adhesion molecule (EpCAM) and cytokeratin (CK) for CTC enumeration and identification, which fails to detect highly aggressive cells that downregulate the expression of CK and EpCAM during the process of epithelial‐mesenchymal transition (EMT).^20^ In addition, non‐tumor epithelial cells derived from inflammation, trauma, and benign epithelial hyperplasia may result in false‐positive results.^21^ As the CellSearch^®^ platform cannot meet clinical requirements, an EpCAM‐independent system integrating negative enrichment with immunostaining and fluorescence in situ hybridization (NE‐imFISH) has been introduced as an alternative option.^13^ The enrichment process of this system involves exclusion of leukocytes by anti‐CD45 antibody and erythrocytes by hemolysis, followed by CTC identification by centromere probe 8 (CEP8) staining, as chromosome 8 polysome is the most common form of chromosome instability observed in PDAC.^22^, ^23^, ^24^, ^25^

In addition to existence in advanced and metastatic stages, CTCs are also widely present in PDAC patients at early stages, suggesting its potential value as a diagnostic tool.^26^, ^27^ It has been reported that CTCs can be an indicator of early diagnosis in pancreatic cancer, liver cancer, breast cancer, prostate cancer, and gynecological cancer.^20^, ^28^, ^29^, ^30^ However, whether CTC enumeration is complementary to CA199 remains largely unknown. In this prospective study, the NE‐imFISH strategy was employed to identify CTCs in 80 PDAC patients and 88 controls, and then we explored the significance of CTC number in combination with CA199 for PDAC diagnosis.

## 2 METHOD

### 2.1 Study design and patients

This prospective, non‐inferior study was conducted in the Affiliated Shengjing Hospital of China Medical University from August 2019 to August 2020. We recruited 80 consecutive patients with newly diagnosed PDAC in this study. Additionally, there were 88 control individuals involved, including 32 consecutive patients with acute pancreatitis, 22 consecutive patients with benign pancreatic masses, and 34 healthy donors randomly selected from the physical examination center. Of the 22 patients with benign pancreatic masses, one had an intraductal papillary mucinous neoplasm, one had a solid pseudopapillary tumor, two had G1 neuroendocrine tumors, and eighteen had cystadenomas. The diagnoses of pancreatic masses were pathologically confirmed by either biopsy or surgical resection, while acute pancreatitis patients were diagnosed by serological and imaging examinations. All the recruited patients had received no cancer‐related therapy or had a history of other tumors before hospitalization, whose clinical data were fully available. The tumor stage was determined according to the 8th edition staging system proposed by the American Joint Committee on Cancer (AJCC).^31^

A 3.2 ml sample of peripheral blood was collected in tubes with ACD‐anticoagulant (Becton Dickinson) for CTC detection, and a 2 ml sample for analysis of CA199. All the blood samples were obtained before any treatments and processed within 12 h after standard venipuncture. Written informed consents were collected from all the participants. This study was conducted in line with the Declaration of Helsinki principles and approved by the local ethics committee with certificate number 2019PS543K.

### 2.2 CTC detection and CA199 measurement

The NE‐imFISH approach developed by Cyttel^®^ was employed to identify CTCs. The enrichment and identification process were based on the standard protocol reported previously.^13^ In short, a 3.2 ml blood sample combined with CS1 buffer (Cyttel Biosciences INC.) was centrifuged at 650 g for 5 min to remove plasma. Then, erythrocytes were lysed with CS2 (Cyttel) for 8 min and removed by another round of centrifugation (650 g for 5 min). After discarding the supernatants and re‐suspending the residual cells with CS1 buffer, CS3 buffer (Cyttel) and magnetic beads (Cyttel) were added to remove most of the leukocytes by magnetic separation and gradient centrifugation (300 g for 5 min). The sedimented cells were fixed on the slides and dried at room temperature for CTC identification.

Afterward, the slides were dehydrated in ethanol and hybridized with CEP8 (Cyttel). Subsequently, slides were incubated with anti‐CD45 conjugated to Alexa Fluor 594 (Cyttel) and mounted with mounting media containing DAPI (Vector Laboratories). The results were read by fluorescence microscope (Nikon Ci). The whole detection procedure was conducted blindly by two trained authors to ensure the reliability of the detection result. Any disagreement was resolved through discussion with a senior technician. CTCs were characterized as CD45−/ DAPI+/CEP8 ≥3.

CA199 in blood samples was measured using a chemiluminescence assay kit (Roche). Levels <37 U/ml were considered as negative.

### 2.3 Sample size calculation and statistical analysis

This study utilized a non‐inferiority design. On the basis of reviewing the literature on CA199 and CTCs identified by NE‐imFISH, we estimated the sensitivity/specificity of CA199 and CTCs were 75%/80% and 83%/86%, respectively.^21^, ^23^, ^32^, ^33^, ^34^ As we only focused on the non‐inferiority rather than the equivalence, the sample size calculation was based on a one‐tailed alpha of 0.025 with a power of 0.8. The non‐inferiority margin is set at 0.1. This calculation required 80 and 86 participants for PDAC and control group, respectively. In the end, we enrolled 80 PDAC patients and 88 controls.

Correlations between clinicopathological data and CTCs were analyzed with chi‐square or Fisher exact tests, and Mann–Whitney U test, as appropriate. Binary logistic regression was applied to evaluate the combined effect of two biomarkers for PDAC diagnosis when coefficients of models were statistically significant. Receiver operating characteristics (ROC) analyses were conducted to evaluate the areas under the curves (AUCs), sensitivity (SN), specificity (SP), positive predictive values (PPVs), and negative predictive values (NPVs). The optimal cut‐off value was determined according to the maximum value of Youden J index (J = SN + SP − 1). Delong tests were used to compare the AUCs of different biomarkers. All 95% confidence intervals (95% CIs) were estimated using the Clopper–Pearson method. Data computation was performed with Stata SE 16.1 (Stata Corp.) and MedCalc 19.5.6 (MedCalc Software bv). A two‐sided *p*‐value of <0.05 was considered statistically significant. All graphs were plotted utilizing GraphPad Prism 8.4 (GraphPad Software) and the “ggalluvial” R package.

## 3 RESULTS

### 3.1 Identification and classification of CTCs

**FIGURE 1:**
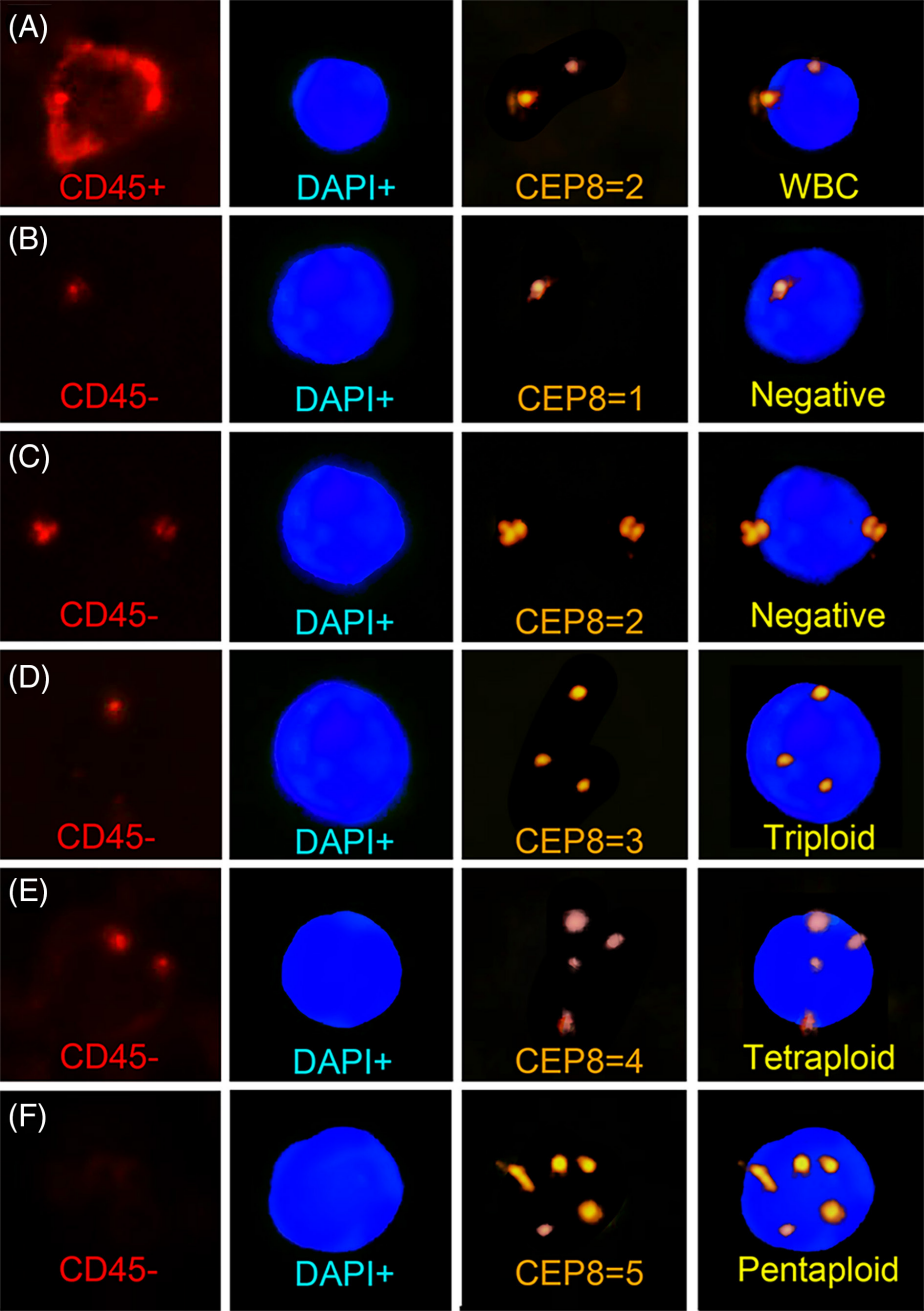
Identification of CTCs by NE‐imFISH strategy. CD45: red; DAPI: blue; CEP8: orange. (A) CD45‐positive cells were regarded as white blood cells. (B, C) CD45‐negative cells with one or two CEP8 signals were considered as negative. (D, E, F) CD45‐negative cells with three or more CEP8 signals were identified as triploid, tetraploid, pentaploid CTCs according to the copy number of chromosome 8

Cells enriched from blood samples were identified and classified according to the leukocyte marker (CD45), cell nucleus marker (DAPI), and chromosome ploidy (CEP8). Negative cells were stained as CD45+/DAPI+/CEP ≥1 (Figure 1A) and CD45–/DAPI+/CEP8 ≤2 (Figure 1B,C), while CTCs were characterized by CD45–/DAPI+/CEP8 ≥3. In our study, cells with more than five CEP8 signals were collectively referred to as multiploidy CTCs because of their rarity. Thus, detected cancer cells were categorized as triploid CTCs for three copies of chromosome 8 (Figure 1D), tetraploid for 4 (Figure 1E), and multiploid for five or more (Figure 1F). As different kinds of CTCs may present in one sample, CTC subtype is used to indicate the number of karyotypes.

### 3.2 Distributions of CA199 and CTCs

**FIGURE 2:**
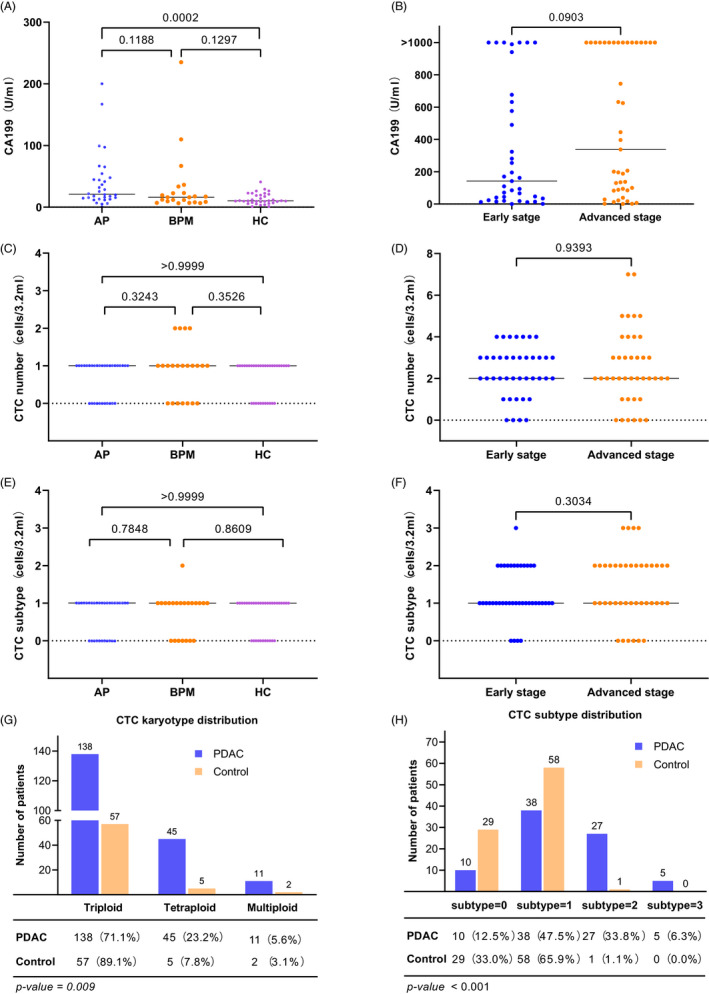
Distributions of CA199 and CTCs in PDAC patients and controls. (A, C, E) The distributions of CA199, CTC number, and CTC subtype among acute pancreatitis (AP), benign pancreatic masses (BPM), and health control (HC). (B, D, F) The distributions of CA199, CTC number, and CTC subtype between early‐stage (stageⅠ–Ⅱ) and late‐stage (stage Ⅲ–Ⅳ) PDAC patients. (G) The karyotype distribution of PDAC patients was significantly different from that of controls. (H) A significant difference was observed in CTC subtype distribution between PDAC patients and controls

The distributions of CA199 and CTCs in different groups were first explored. In the control group, CTC subtype and CTC number were not significantly different among patients with acute pancreatitis, patients with benign pancreatic masses, and health controls (Figure 2C,E). As for CA199, statistical significance was only found between the comparison between health controls and acute pancreatitis (*p* = 0.0002; Figure 2A). Similarly, CA199, CTC subtype, and CTC number could not distinguish between early‐stage (stage Ⅰ–Ⅱ) and advanced‐stage (stage Ⅲ–Ⅳ) patients (Figure 2B,D,F). Given the within‐group similarities, in the subsequent analyses, we only divided enrolled participants into PDAC group and control group.

As illustrated in Figure 2G, CTC karyotype distribution between the two groups was significantly different (*p* = 0.009). A total of 194 CTCs were detected in 70 in 80 PDAC patients (mean number, 2.43 CTCs/3.2 ml), consisting of 138 (71.1%) triploid cells, 45 (23.2%) tetraploid cells, 11 (5.7%) multiploid cells. Aneuploidy can also present in non‐tumor patients. Within 64 CTCs detected in 59 of 88 non‐malignant controls (mean number, 0.72 CTCs/3.2 ml), 57 (89.1%) were triploid cells, 5 (7.8%) were tetraploid cells, and 2 (3.1%) were multiploid cells. Two conclusions are evident from these data. First, the frequency of one kind of karyotype decreases with the increasing copy number of chromosome 8. Second, tetraploid and polyploid cells are rarely seen in the control group, mainly in cancer patients.

Statistical significance between the two groups was also shown in the composition of CTC subtype number (*p* < 0.001). The average number of CTC subtype was 1.31 in the PDAC group and 0.68 in the control group. Of the 88 control subjects, only one was detected to have two subtypes. Comparatively, 32 (40.1%) PDAC patients tended to show mixed subtypes with two or three karyotypes (Figure 2H).

### 3.3 Comparison of the diagnostic performance among CTCs, CA199, and the dual‐marker panel

**FIGURE 3:**
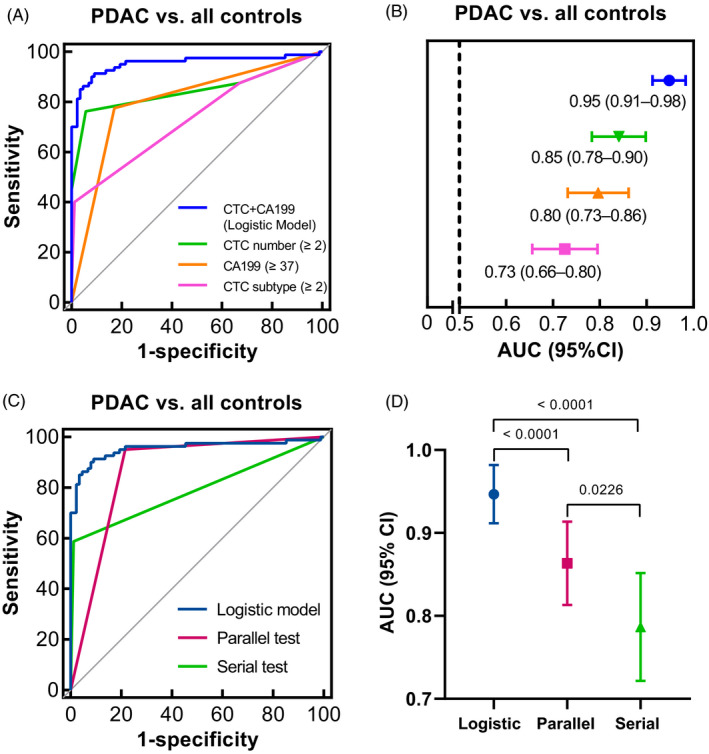
Assessment of the diagnostic significance of CTC number in combination with CA199. (A, B) ROC curves and AUC analyses for CA199, CTC number, CTC subtype, and the logistic model. (C, D) ROC curves and AUC analyses for three methods integrating CA199 and the CTC number, including logistic model, parallel and serial test algorithms

ROC curves and AUC analyses were conducted to assess diagnostic abilities in distinguishing PDAC cases from controls (Table 1; Figure 3A,B). According to the Youden index, the cut‐off value for CTC number was 2 CTCs/3.2 ml peripheral blood, yielding a sensitivity of 0.76 (0.65–0.85) and a specificity of 0.94 (0.87–0.98). At the optimal cut‐off value of 2 subtypes/3.2 ml, the sensitivity and specificity of CTC subtype were 0.40 (0.29–0.52) and 0.99 (0.94–1.00), respectively. As shown in Table 2, we further examined the associations between CTCs and clinicopathological data. The positive rates of CTC number and CTC subtype did not correlate with age, sex, tumor location, tumor differentiation, T stage, N stage, M stage, TNM stage, and serum CA199 expression level (<37 or ≥37 U/ml).

**TABLE 1:** Diagnostic performances of CTC, CA199, and the combination of the two markers

Group	Cut‐off value	AUC (95% CI)	*p‐*value of AUC	SN (95% CI)	SP (95% CI)	PPV (95% CI)	NPV (95% CI)	Delong test *p‐*value	
								vs. CA199	vs. CTC num.
CA199 + CTC	>0.25^a^ (Logistic)	0.95 (0.91–0.98)	<0.0001	0.91 (0.83–0.96)	0.91 (0.83–0.96)	0.90 (0.83–0.95)	0.92 (0.85–0.96)	<0.0001	0.0002
CA199 + CTC	Serial^b^	0.79 (0.72–0.85)	<0.0001	0.59 (0.47–0.70)	0.99 (0.94–1.00)	0.98 (0.87–1.00)	0.73 (0.67–0.77)	0.6294	0.0370
CA199 + CTC	Parallel^c^	0.87 (0.81–0.91)	<0.0001	0.95 (0.88–0.99)	0.78 (0.68–0.87)	0.80 (0.73–0.86)	0.95 (0.87–0.98)	0.0072	0.5552
CTC number	≥2	0.85 (0.78–0.90)	<0.0001	0.76 (0.65–0.85)	0.94 (0.87–0.98)	0.92 (0.84–0.97)	0.81 (0.75–0.87)	0.3360	1.00
CA199	≥37	0.80 (0.73–0.86)	<0.0001	0.78 (0.67–0.86)	0.83 (0.73–0.90)	0.81 (0.72–0.87)	0.80 (0.73–0.86)	1.00	0.3360
CTC subtype	≥2	0.73 (0.66–0.80)	<0.0001	0.40 (0.29–0.52)	0.99 (0.94–1.00)	0.97 (0.82–1.00)	0.64 (0.60–0.69)	0.1264	<0.0001

Abbreviations: AUC, area under the curves; CTC, circulating tumor cell; NPV, negative predictive value.PPV, positive predictive value; SN, sensitivity; SP, specificity.

Blue coloured font indicates to explain the three methods of joint diagnosis.

^a^ The logistic score = 0.0157 × CA199 level + 2.2624 × CTC number − 4.4098.

^b^ The positive result of serial test indicated CA199 and CTC number were both positive.

^c^ The positive result of parallel test indicated at least one was positive between CA199 and CTC number.

**TABLE 2:** The relationships between clinicopathological characteristics and positive rate of CTC number and CTC subtype

Characteristics	CTC number		*p*‐value	CTC subtype		*p*‐value
	<2	≥2		<2	≥2	
Age						
<60	10	27	0.52	24	13	0.41
≥60	9	34		24	19	
Sex						
Male	9	42	0.09	27	24	0.09
Female	10	19		21	8	
Location						
Body/Tail	5	20	0.60	15	10	>0.99
Head/Neck	14	41		33	22	
Differentiation						
High	6	7	0.18	10	3	0.32
Moderate	4	24		15	13	
Poor	2	10		9	3	
Uncertain	7	20		15	13	
Tumor size						
T1–T2	12	26	0.12	26	12	0.14
T3–T4	7	35		22	20	
Lymph nodes						
N0	9	28	0.56	25	13	0.58
N1	2	14		10	6	
N2	1	2		2	1	
Nx	7	16		11	12	
Metastasis						
No	13	50	0.22	41	22	0.07
Yes	6	11		7	10	
TNM stage						
ⅠA–ⅡB	9	31	0.79	27	13	0.17
Ⅲ–Ⅵ	10	30		21	19	
CA199 (U/ml)						
<37	4	14	>0.99	11	7	0.91
≥37	15	47		37	25	

We transformed the serological levels of CA199 into 0 or 1 values according to the cut‐off value of 37 U/ml, which was commonly used in clinical practice. Thus, the AUC value could reliably and directly reflect the diagnostic performance of CA199 at this cut‐off point. As expected, no statistical difference was found between CTC number and CA199 in the AUC comparison (AUC_CTC number_ = 0.85, AUC_CA199_ = 0.80, respectively; Delong test *p* = 0.3360). However, as single markers, the performance of CTC subtype was significantly inferior to CTC number (AUC_CTC subtype_ = 0.73; Delong test *p*_vs_. _CTC number_ < 0.0001).

Next, we utilized three methods to assess the diagnostic performance of CA199 in combination with CTC number, including logistic regression, parallel and serial algorithms (Tables 1, S1; Figure 3A–D). Compared with CA199, the parallel test had significantly better performance in diagnosis instead of serial test (AUC_parallel_ = 0.87, Delong test *p*_vs_. _CA199_ = 0.0072; AUC_serial_ = 0.79, Delong test *p*_vs_. _CA199_ = 0.6294). The logistic model could further enhance the diagnostic ability of parallel test (AUC_logistic_ = 0.95, Delong test *p*_vs serial_ < 0.0001). This dual‐marker logistic model improved the sensitivity of CA199 from 0.78 to 0.91 accompanied by 0.08 rise in specificity and the sensitivity of CTC number from 0.76 to 0.91 at the cost of 0.03 reduction in specificity (Delong test *p*_vs CA199_ < 0.0001, *p*_vs CTC number_ = 0.0002). Collectively, our results demonstrated that CTC number could complement CA199 in differentiating PDAC cases from controls.

### 3.4 Subgroup analysis

To investigate why CTC number was complementary to CA199, we evaluated the performances of 3 individual markers in different subgroups.

**FIGURE 4:**
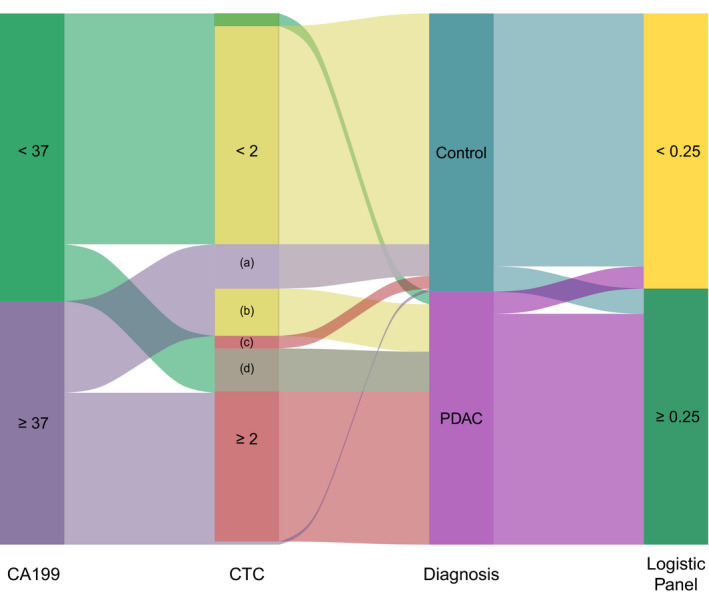
The alluvial plot depicting the results of CA199, CTC number, and the dual‐marker panel based on logistic regression. Area (A) represents the false‐positive results of CA199 that can be correctly classified by CTC. Area (B) represents the false‐negative results of CTC that can be correctly classified by CA199. Area (C) represents the false‐positive results of CTC that can be correctly classified by CA199. Area (D) represents the false‐negative results of CA199 that can be correctly classified by CTC

(1) Subgroup analysis by CA199 (Table 3). One of the main shortcomings of CA199 as the biomarker for PDAC is the limited sensitivity, such as false‐negative results observed in 5%–10% of people with Lewis antigen‐negative genotype.^35^ Because of this, we divided our participants into CA199 low (CA199 < 37 U/ml) and high (CA199 ≥ 37 U/ml) groups. In the low expression group, 18 PDAC patients were falsely diagnosed as negative by CA199. CTC number could correctly identify 14 of them while maintaining high specificity (sensitivity = 0.78; specificity = 0.95; Figure 4D). False‐positive results were observed in 15 controls, including 11 patients with acute pancreatitis, of which 14 could be correctly classified by CTC number (sensitivity = 0.76; specificity = 0.93; Figure 4A). Therefore, CTC number could complement CA199 in determining the diagnosis of PDAC.

**TABLE 3:** Subgroup analysis by CA199 and CTC number

Subgroup	AUC (95% CI)	*p*‐value	Cut‐off value	SN (95% CI)	SP (95% CI)	PPV (95% CI)	NPV (95% CI)
CA199 < 37 U/ml (PDAC:Control = 18:73)							
CTC number	0.86 (0.77–0.92)	<0.0001	≥2	0.78 (0.52–0.94)	0.95 (0.87–0.99)	0.78 (0.57–0.90)	0.95 (0.88–0.98)
CTC subtype	0.73 (0.63–0.82)	0.0003	≥2	0.39 (0.17–0.64)	0.99 (0.93–1.00)	0.88 (0.48–0.98)	0.87 (0.82–0.91)
CA199 ≥ 37 U/ml (PDAC:Control = 62:15)							
CTC number	0.84 (0.74–0.92)	<0.0001	≥2	0.76 (0.63–0.86)	0.93 (0.68–1.00)	0.98 (0.88–1.00)	0.48 (0.37–0.60)
CTC subtype	0.74 (0.62–0.83)	<0.0001	≥2	0.40 (0.28–0.54)	1.00 (0.78–1.00)	1.00	0.21 (0.20–0.22)
CTC number <2 CTCs/3.2 ml (PDAC:Control = 19:83)							
CA199	0.81 (0.77–0.92)	<0.0001	≥37	0.79 (0.54–0.94)	0.83 (0.73–0.91)	0.51 (0.39–0.65)	0.94 (0.88–0.98)
CTC number ≥2 CTCs/3.2 ml (PDAC:Control = 61:5)							
CA199	0.79 (0.66–0.88)	0.0059	≥37	0.77 (0.65–0.87)	0.80 (0.28–1.00)	0.98 (0.89–1.00)	0.22 (0.13–0.35)

Abbreviations: AUC, area under the curves; CTC, circulating tumor cell; NPV, negative predictive value; PPV, positive predictive value; SN, sensitivity; SP, specificity.

(2) Subgroup analysis by CTC number (Table 3). A total of 19 patients were wrongly diagnosed as CTC‐negative (<2 CTCs/ 3.2 ml), while CA199 could correctly identify 15 of them while maintaining high specificity (sensitivity = 0.79; specificity = 0.83; Figure 4B). In addition, only five patients showed false‐positive results of CTC number, of which 4 patients could be correctly differentiated by CA199 (sensitivity = 0.77; specificity = 0.80; Figure 4C). These findings demonstrated that CA199 could also complement CTCs in the diagnosis of PDAC.

## 4 DISCUSSION

PDAC is the deadliest cancer with a dismal prognosis, which can be improved through early diagnosis. To date, CA199 is the routinely used serum biomarker for PDAC management with several unfavorable drawbacks.^32^, ^33^ Thus, there is an urgent need to establish a more accurate diagnostic system. As a non‐invasive approach harboring the great potential to compensate for the shortcomings of CA199, CTC detection, also known as liquid biopsy, has remarkable value in diagnosis, prognosis, as well as evaluation of tumor metastasis and relapse.^20^

CTCs are cancer cells shed into the peripheral bloodstream either from primary or from metastatic tumors.^12^ Several techniques have been introduced to identify CTCs in PDAC patients, mainly based on physical properties (including ScreenCell^®^, ISET^®^, density gradient separation) or surface antibody features (including CellSearch^®^, NE‐imFISH, Nano Velcro Chip) of cells. The EpCAM‐dependent CellSearch^®^ platform is the standard method approved by FDA for CTC detection. However, this strategy fails to identify a large subgroup of CTCs with low EpCAM expression, which is downregulated during the process of EMT.^20^ It is, therefore, not surprising that the detection rates reported by previous studies are undesirable, ranging between 21% and 45%.^14^, ^15^, ^16^, ^17^, ^18^, ^19^, ^20^ Other studies which used ScreenCell^®^, ISET^®^, Nano Velcro Chip techniques achieved more CTC‐positive results (68%, 78%–90%, 78%, respectively) compared with the CellSearch^®^ platform,^36^, ^37^, ^38^, ^39^ while some studies only reported a detection rate of 34% after density gradient separation.^40^

In our research, we employed a negative enrichment strategy followed by CD45 staining and FISH with CEP8 for CTC identification, which could also detect CK‐negative or EpCAM‐negative CTCs. Hyperdiploid cells without CD45 signals were regarded as CTCs. This strategy was validated by previous studies. Han et al. used this technique to identify CTCs from 95 PDAC patients and 48 health controls with 2 CTCs/3.2 ml as the optimal cut‐off point, reaching 76% sensitivity and 69% specificity.^23^ Ning et al. enriched CTCs from 30 patients with pulmonary cancer. When the cut‐off value was 2 CTCs/3.2 ml, the sensitivity and specificity of this strategy were 83% and 98.6%, respectively.^21^ Gao et al. combined the staining of CD45, CK, DAPI, and CEP8‐FISH for CTC enumeration in 25 PDAC patients and 25 healthy donors, yielding a sensitivity of 88% and a sensitivity of 90%. Notably, CK+/CD45−/DAPI+/CEP8 = 2 was also defined as CTCs. Among the 103 CTCs detected in their study, only 1 was CK‐positive with two CEP8 signals, demonstrating the CK could not contribute to the improvement of detection rate. In the present studies, 61 of 80 (76%) patients were detected to be CTC‐positive at the cut‐off value of 2 CTCs/3.2ml, while 83 of 88 (94%) controls were CTC‐negative. Furthermore, the NE‐imFISH method requires less blood (3.2 ml) compared with CellSearch^®^ (7.5 ml), Nano Velcro Chip (4 ml), ScreenCell^®^ (6 ml), ISET^®^ (5–10 ml), density gradient separation system (20 ml).^15^, ^36^, ^37^, ^38^, ^39^, ^40^ Our findings, combined with those of other studies, suggest that the NE‐imFISH strategy that integrated negative enrichment with immunostaining and fluorescence in situ hybridization is feasible for CTC detection.

As far as we know, this study is the first to combine CA199 and CTC identified by the NE‐imFISH method for the diagnosis of PDAC. The sensitivity and specificity of CA199 in our study reached 0.80 and 0.78. No significant difference was found in the comparison with AUCs between CTC number and CA199 (Delong test *p* = 0.3360), indicating that CTCs are comparable to CA199 as a diagnostic marker. Of note, the dual‐marker model based on logistic regression outperformed both CTC number and CA199 alone (Delong test *p*_vs. CA199_ < 0.0001, *p*_vs_. _CTC number_ = 0.0002), exhibiting higher sensitivity (91%) and specificity (91%). This significant improvement was attributed to the complementary role between the two markers. When CA199 showed false‐positive results in acute pancreatitis and false‐negative results in Lewis antigen‐negative patients, CTCs could more accurately differentiate PDAC from non‐malignancy. Similarly, CA199 was able to partially correct the false‐negative diagnoses of CTCs. Taken together, our study suggests that the combination of CTC number and CA199 is a promising predictor for PDAC diagnosis.

Han et al. pointed out that CTC subtype could be used as a diagnostic marker with a sensitivity of 54% and a specificity of 85% using 2 subtypes/3.2 ml as the cut‐off value.^23^ In this study, the optimal cut‐off value of CTC subtype was the same, achieving 40% sensitivity and 98.9% specificity. As the cut‐off values for both CTC subtype and CTC number were two, the CTC number must be positive when the CTC subtype was positive, but not necessarily vice versa. Therefore, CTC subtype was significantly inferior to CTC number (Delong test *p* < 0.0001) and not suggested for PDAC diagnosis.

CTCs play a crucial role in initiating metastasis in distant organs and significantly correlate with prognosis in patients with lung, breast, prostate, and colorectal cancer.^4^ In addition, CTCs derived from PDAC patients can also survive in mouse models and form metastasis.^41^ However, our study demonstrated that CTC number and subtype in PDAC patients were not associated with tumor size, distant metastasis, lymph node metastasis, and TNM stage, which was consistent with previous studies.^23^, ^34^ Although tumors can release thousands of CTCs daily into circulation, the vast majority of CTCs have limited persistence in the harsh physical and immunological environment of the blood.^42^ For example, the half‐life of CTC is only 1–2.4 h in patients with breast cancers.^43^ Additionally, most cells cannot adapt to the new microenvironment at a distant site. As a result, the whole process was highly inefficient, with only about 1%–4% of CTCs successfully forming metastatic foci.^43^, ^44^, ^45^ Moreover, the metastasis of tumors depends not only on the number of CTCs in the circulation but also on their gene expression characteristics, which are dynamically heterogeneous during tumor evolution, thus forming CTC subgroups with different invasive abilities.^12^ Therefore, it is difficult to predict the degree of tumor progression only from the result of CTC number.

Aneuploidy is the most common feature of solid tumors caused by chromosomal instability.^46^, ^47^ Notably, it can also exist in normal somatic cells, such as stem cells, cardiomyocytes, hepatocytes, and skeletal muscle cells.^13^, ^48^ When in a state of stress, injury, and other pathological conditions, these cells will fuse into polyploid cells to lengthen their lifespans and enhance the stability of cellular function.^48^ Theoretically, CTCs should not be present in non‐malignant diseases. As our CTC detection approach was based on the aneuploidy of chromosome 8, the cells identified as CTCs in the control group were most probably cells with abnormal metabolism instead of real tumor cells. Nevertheless, the frequency of aneuploid cells was much lower in the control group.

In conclusion, the current data support CTC number identified by the NE‐imFISH method is a feasible biomarker comparable to CA199 in detecting PDAC. The dual‐marker panel consisting of CA199 and CTC number can significantly improve upon the diagnostic performance of CA199 alone, highlighting the promising clinical utilization as a convenient and effective strategy for PDAC surveillance. However, our study was conducted in a single center with limited sample size. Further clinical studies with more participants are warranted to verify our findings.

## CONFLICT OF INTEREST

The authors have no conflicts of interests to declare.

## AUTHOR CONTRIBUTIONS

Xiaodong Tan and Junliang Chen conceived and planned this experiment. Zhihao Liu and Junliang Chen carried out the experiments of CTC detection. Huaitao Wang and Lei Zhou collected clinicopathological data of patients. Xiaodong Tan, Junliang Chen, Zhihao Liu, Huaitao Wang, and Lei Zhou contributed to the interpretation of results. Junliang Chen took the lead in writing the manuscript. All authors provided vital feedback to shape the final version of the manuscript.

## Supporting information

Table S1

## Data Availability

The data that support the findings of this study are available on request from the corresponding author, XD Tan.
