# Supplementary material for: A combination of circulating tumor cells and CA199 improves the diagnosis of pancreatic cancer
Source: J Clin Lab Anal. 2022 Mar 25;36(5):e24341. doi: 10.1002/jcla.24341 (PMC9102772; doi:10.1002/jcla.24341)
Supplement: Supplementary file 1 — Table S1 [file JCLA-36-e24341-s001.docx]

| **Variable** | **Coefficient** | **Std. Error** | **Wald** | **P** |
| --- | --- | --- | --- | --- |
| CA199 | 0.0157 | 0.0042530 | 13.5600 | 0.0002 |
| CTC number | 2.2624 | 0.44192 | 26.2093 | <0.0001 |
| Constant | -4.4098 | 0.70556 | 39.0639 | <0.0001 |

**Table S1:** The results of logistic regression model.
